# Effects of Different Missing Data Imputation Techniques on the Performance of Undiagnosed Diabetes Risk Prediction Models in a Mixed-Ancestry Population of South Africa

**DOI:** 10.1371/journal.pone.0139210

**Published:** 2015-09-25

**Authors:** Katya L. Masconi, Tandi E. Matsha, Rajiv T. Erasmus, Andre P. Kengne

**Affiliations:** 1 Division of Chemical Pathology, Faculty of Health Sciences, National Health Laboratory Service (NHLS) and University of Stellenbosch, Cape Town, South Africa; 2 Non-Communicable Diseases Research Unit, South African Medical Research Council, Cape Town, South Africa; 3 Department of Biomedical Technology, Faculty of Health and Wellness Sciences, Cape Peninsula University of Technology, Cape Town, South Africa; 4 Department of Medicine, University of Cape Town, Cape Town, South Africa; University of Tolima, COLOMBIA

## Abstract

**Background:**

Imputation techniques used to handle missing data are based on the principle of replacement. It is widely advocated that multiple imputation is superior to other imputation methods, however studies have suggested that simple methods for filling missing data can be just as accurate as complex methods. The objective of this study was to implement a number of simple and more complex imputation methods, and assess the effect of these techniques on the performance of undiagnosed diabetes risk prediction models during external validation.

**Methods:**

Data from the Cape Town Bellville-South cohort served as the basis for this study. Imputation methods and models were identified via recent systematic reviews. Models’ discrimination was assessed and compared using C-statistic and non-parametric methods, before and after recalibration through simple intercept adjustment.

**Results:**

The study sample consisted of 1256 individuals, of whom 173 were excluded due to previously diagnosed diabetes. Of the final 1083 individuals, 329 (30.4%) had missing data. Family history had the highest proportion of missing data (25%). Imputation of the outcome, undiagnosed diabetes, was highest in stochastic regression imputation (163 individuals). Overall, deletion resulted in the lowest model performances while simple imputation yielded the highest C-statistic for the Cambridge Diabetes Risk model, Kuwaiti Risk model, Omani Diabetes Risk model and Rotterdam Predictive model. Multiple imputation only yielded the highest C-statistic for the Rotterdam Predictive model, which were matched by simpler imputation methods.

**Conclusions:**

Deletion was confirmed as a poor technique for handling missing data. However, despite the emphasized disadvantages of simpler imputation methods, this study showed that implementing these methods results in similar predictive utility for undiagnosed diabetes when compared to multiple imputation.

## Background

Missing data is common in predictive research, and can negatively affect the performance of risk prediction models. In an ideal setting, a subject with missing data on a predictor or outcome variable should be replaced with a randomly selected subject from the source population. However, replacement is burdensome and most often impossible. Instead, researchers can use observed data to make an estimation of the status of the participants for the characteristic with missing value. Imputation techniques are based on the basic principle of replacement, indicating that any conclusion drawn from the study should not depend on the sample that is involved in the study. Should each subject in the chosen sample be replaced by a new subject from the same source population as the original subject, the conclusions should not be compromised [1].

It is widely advocated that imputation of missing data is superior to the overlooking of the missing data, that the indicator method often provides biased results, that conditional mean imputation is better than unconditional implementation, and that multiple imputation method is better than single imputation [1–13]. However, studies have suggested that simple methods for filling missing data can be just as accurate as complex methods, allowing for easier implementation in prediction studies [14, 15]. The type and percentage of missing data are important determining factors for the accuracy of the different imputation methods. Data missing completely at random (MCAR) has a low probability that the observation missing is related to any other patient characteristics and most simple techniques for handling missing data give unbiased results [4]. When the missing data depends on information that is not observed, the missing data is considered missing not at random (MNAR) [3]. Although there is no advocated method available to handle the valuable information that has been lost through MNAR data, multiple imputation can be unbiased for MNAR data [2]. Most often, missing data are neither MCAR nor MNAR [11], but rather missing at random (MAR). This type of missing data is missing at random conditional on the individuals other characteristics that are available at the time of analysis [3]. When missing data are MAR, common and simple techniques used to handle missing data such as complete case and available case analysis, indicator method and overall mean imputation are likely to introduce selection bias as the database is no longer a random sample of the source population [5, 6, 11, 16].

This study aims to implement a number of simple and more complex imputation methods for filling missing data, and assess the comparative effects on the performance of undiagnosed diabetes risk prediction models during external validation. For this purpose, we use data for mixed-ancestry South African who took part in the Bellville-South study in Cape Town.

## Material and Methods

### Database

Details of the study design and recruitment of the database that served as the basis for all imputation methods implementation have been described below. The Bellville South Study was a cross-sectional study conducted from mid-January 2008 to March 2009 (cohort 1), and from January 2011 to November 2011 (cohort 2). The study was approved by the Ethics Committee of the Cape Peninsula University of Technology (CPUT/HW-REC 2008/002 and CPUT/HW-REC 2010) and Stellenbosch University (N09/05/146). Recruited subjects were visited by recruitment team the evening before participation and reminded of all the survey instructions. All participants signed written informed consent after all the procedures had been fully explained in the language of their choice.

### Research setting

Bellville-South is located within the Northern suburbs of Cape Town, South Africa and is a traditionally a Coloured township formed in the late 1950s. According to the 2011 population census, its population stands at approximately 29 301 with 76.0% (22 270) consisting of the mixed ancestry individuals [17, 18]. The target population for this study were subjects between the ages of 35 and 65 years and their number was estimated to be 6 500 in the 2001 population census [19].

### Research Design and Study Population

The data was collected during January 2008 to March 2009. Using a map of Bellville South, multistage stratified random sampling was approached as follows: From a list of streets from each stratum, the streets were then classified as short, medium and long streets based on the number of houses. Streets with houses ≤ 22 were classified as short, medium; houses 23–40 and long streets were > 40 houses. A total of 16 short streets representing approximately 190 houses, 15 medium streets representing approximately 410 houses and 12 long streets representing approximately 400 houses were randomly selected across the different strata. From the selected streets, all household members meeting the selection criteria were invited to participate in the study. Community authorities requested that participants outside the random selection area should benefit from the study.

### Recruitment Strategy

Information regarding the project was disseminated to the local residents through the local radio station, community newspaper, brochures and fliers; the latter bearing information about the project and distributed through school children and taxis to the local residents by the recruitment team. Recruited subjects were visited by the recruitment team the evening before participation and reminded of all the survey instructions.

### Data collection

A detailed protocol describing data-collection procedures (questionnaires and physical examination) was developed. The questionnaire designed to retrospectively obtain information on lifestyle factors such as smoking and alcohol consumption, physical activity, diet, family history of CVD and DM, and demographics was administered by trained personnel. A detailed drug history was obtained by interrogation and by examining the clinic cards as well as the record of drugs that participants brought to the study site. Clinical measurements included height, weight, hip and waist circumferences, body fat measurements and blood pressure.

### Diabetes diagnosis

All participants, except the self-reported diabetic subjects, confirmed by either medical card record or drugs in use, had blood taken for fasting blood glucose and underwent a 75 g oral glucose tolerance test (OGTT) as prescribed by the WHO. Diabetes was diagnosed according to the WHO 2006 criteria [20].

### Identification of undiagnosed diabetes prediction models

Existing prediction models were obtained from a systematic review by Brown *et al*, 2012 [21]. Models met the criteria for model selection for this paper if they were developed to predict the presence of undiagnosed diabetes based on predictors measured in the Bellville South study. We focused on models developed from non-invasively measure predictors. Therefore the models retained were: Cambridge Risk model [22], Kuwaiti Risk model [23], Omani Diabetes Risk model [24], Rotterdam Predictive model 1 [25] and the simplified Finnish Diabetes Risk model [26]. Model characteristics and formulas have been published by Masconi *et* al [27]. All models included age as a predictor, while a range of other predictors were variably combined in models. These included: sex, BMI, use of antihypertensive medication, family history of diabetes, waist circumference, past or current smoking and the use of corticosteroids. Table 1 shows the overview of the performance of the undiagnosed diabetes risk prediction models across the five imputation methods.

**Table 1:** Overview of the performance of the undiagnosed diabetes risk prediction models across the five imputation methods before (original) and after intercept adjustment (adjusted).

Models	Performance measure	Deletion		Simple		Conditional		Stochastic		Multiple	
		Original	Adjusted	Original	Adjusted	Original	Adjusted	Original	Adjusted	Original	Adjusted
**Cambridge diabetes risk model**											
	E/O (95% CI)	1.81 (1.09; 2.52)	1.22 (0.61; 1.83)	2.07 (1.40; 2.75)	1.28 (0.69–1.87)	2.01 (1.28; 2.75)	1.27 (0.64–1.90)	2.17 (1.41; 2.93)	1.27 (0.64–1.90)	2.16 (1.40; 2.92)	1.30 (0.66–1.94)
	Brier score	0.193		0.181		0.185		0.186		0.189	
	Yates slope	0.379		-1.401		-1.374		-1.399		-1.441	
	C-statistic (95% CI)	0.67 (0.62–0.72)		0.69 (0.65–0.73)		0.68 (0.63–0.72)		0.68 (0.64–0.73)		0.68 (0.64–0.72)	
**Kuwaiti Risk model**											
	E/O (95% CI)	0.72 (0.40; 1.12)	0.94 (0.47–1.41)	0.79 (0.44; 1.14)	0.96(0.51–1.41)	0.79 (0.39; 1.20)	0.96 (0.45–1.47)	0.82 (0.44; 1.20)	0.96 (0.45–1.47)	0.82 (0.42; 1.22)	0.96 (0.55; 1.37)
	Brier score	0.141		0.122		0.126		0.125		0.123	
	Yates slope	0.496		-0.459		-0.514		-0.473		-0.534	
	C-statistic (95% CI)	0.68 (0.63–0.73)		0.70 (0.66–0.74)		0.69 (0.65–0.73)		0.69 (0.65–0.74)		0.69 (0.65–0.73)	
**Omani diabetes risk model**											
	E/O (95% CI)	1.28 (0.63; 1.93)	1.06 (0.47; 1.66)	1.40 (0.82; 1.98)	1.08 (0.56; 1.60)	1.40 (0.75; 2.05)	1.08 (0.50; 1.66)	1.56 (0.81; 2.30)	1.08 (0.50; 1.66)	1.54 (0.77; 2.31)	1.11 (0.51; 1.71)
	Brier score	0.164		0.141		0.149		0.142		0.153	
	Yates slope	0.392		-1.065		-1.104		-1.049		-1.196	
	C-statistic (95% CI)	0.66 (0.61–0.70)		0.67 (0.63–0.71)		0.65 (0.61–0.70)		0.67 (0.63–0.72)		0.65 (0.61–0.69)	
**Rotterdam diabetes risk model**											
	E/O (95% CI)	0.54 (0.50; 1.04)	0.98 (0.91–1.05)	0.65 (0.56; 0.74)	0.99 (0.83–1.14)	0.59 (0.48; 0.71)	0.99 (0.93–1.04)	0.65 (0.57; 0.74)	0.99 (0.93–1.04)	0.65 (0.57; 0.73)	0.99 (0.87–1.11)
	Brier score	0.147		0.126		0.130		0.129		0.127	
	Yates slope	0.971		0.558		0.539		0.535		0.498	
	C-statistic (95% CI)	0.64 (0.59–0.69)		0.65 (0.61–0.70)		0.65 (0.60–0.69)		0.65 (0.60–0.70)		0.65 (0.61–0.70)	
**Simplified Finnish diabetes risk model**											
	E/O (95% CI)	0.26 (0.13; 0.39)	0.89 (0.51; 1.26)	0.34 (0.17; 0.52)	0.92 (0.53; 1.31)	0.34 (0.18; 0.50)	0.92 (0.56; 1.28)	0.35 (0.17; 0.52)	0.92 (0.56; 1.28)	0.35 (0.17; 0.53)	0.92 (0.53;– 1.32)
	Brier score	0.157		0.133		0.136		0.136		0.133	
	Yates slope	0.491		-0.021		0.080		-0.053		-0.045	
	C-statistic (95% CI)	0.67 (0.62–0.71)		0.66 (0.62–0.70)		0.67 (0.63–0.72)		0.66 (0.62–0.70)		0.66 (0.62–0.70)	

### Statistical methods

#### Analysis of missingness

Data analysis used the R statistical software, version 3.1.2 [28]. Aggregation plots were created using the ‘*VIM’* package to identify of the pattern of missing data for each variable. The corresponding frequencies were tabulated.

#### Identification of imputation methods

A comprehensive search was previously carried out on the imputation methods available [29]. The aim was to compare deletion, single and multiple imputation techniques. To allow for a broad spectrum of techniques, it was decided to compare pair-wise deletion [30], simple imputation [31], conditional mean imputation [7, 30], stochastic regression [7, 32] and multiple imputation for non-monotone missing patterns [16]. Imputation was completed on the outcome and all variables. Where applicable, the outcome and all variables were used as a predictor for the variable being imputed.

#### Imputation

*Simple imputation* via mean substitution was implemented with the package ‘*Hmisc*’ through the function ‘impute (x, fun = mean)’ where x is the predictor of interest [33]. *Conditional mean imputation* was implemented through the creation of a regression model and the subsequent inbuilt ‘*predict*’ function. Imputation via *stochastic regression* used the method ‘*norm*.*nob’* of the R package ‘*mice* [34]. *Multiple imputation for non-monotone missing patterns* via the Multiple Imputation by Chained Equations (MICE) method, using fully conditional specification (FCS) was implemented using the ‘*mice*’ package [34]. The *m* imputed datasets were analysed separately, then the estimates and the associated variance from the imputed data sets combined using rules established by Rubin that incorporates the within and between imputation variability [6].

#### Model performance

The original selected models were validated for the overall data and subsets using the formulas, both prior to recalibration and following intercept adjustment to eliminate differences in diabetes prevalence between the development population of the model and this test population. The predicted probability of undiagnosed diabetes for each participant was computed using the baseline measured predictors. The performance was expressed in terms of discrimination and calibration. Discrimination describes the ability of the model’s performance in distinguishing those at a high risk of developing diabetes from those at low risk [35]. The discrimination was assessed and compared using concordance (C) statistic and non-parametric methods [36].

Calibration describes the agreement between the probability of the outcome of interest as estimated by the model, and the observed outcome frequencies [30]. It was assessed with formal statistical tests, determining the agreement between the expected (E) and observed (O) rates (E/O). The 95% confidence intervals for the expected/observed probabilities (E/O) ratio were calculated assuming a Poisson distribution [37]. We also calculated 1) the Yates slope, which is the difference between mean predicted probability of type 2 diabetes for participants with and without prevalent undiagnosed diabetes, with higher values indicate better performance; and 2) the Brier score, which is the squared difference between predicted probability and actual outcome for each participant with values ranging between 0 for a perfect prediction model and 1 for no match in prediction and outcome [30, 35].

## Results

### Data available

**Fig 1:**
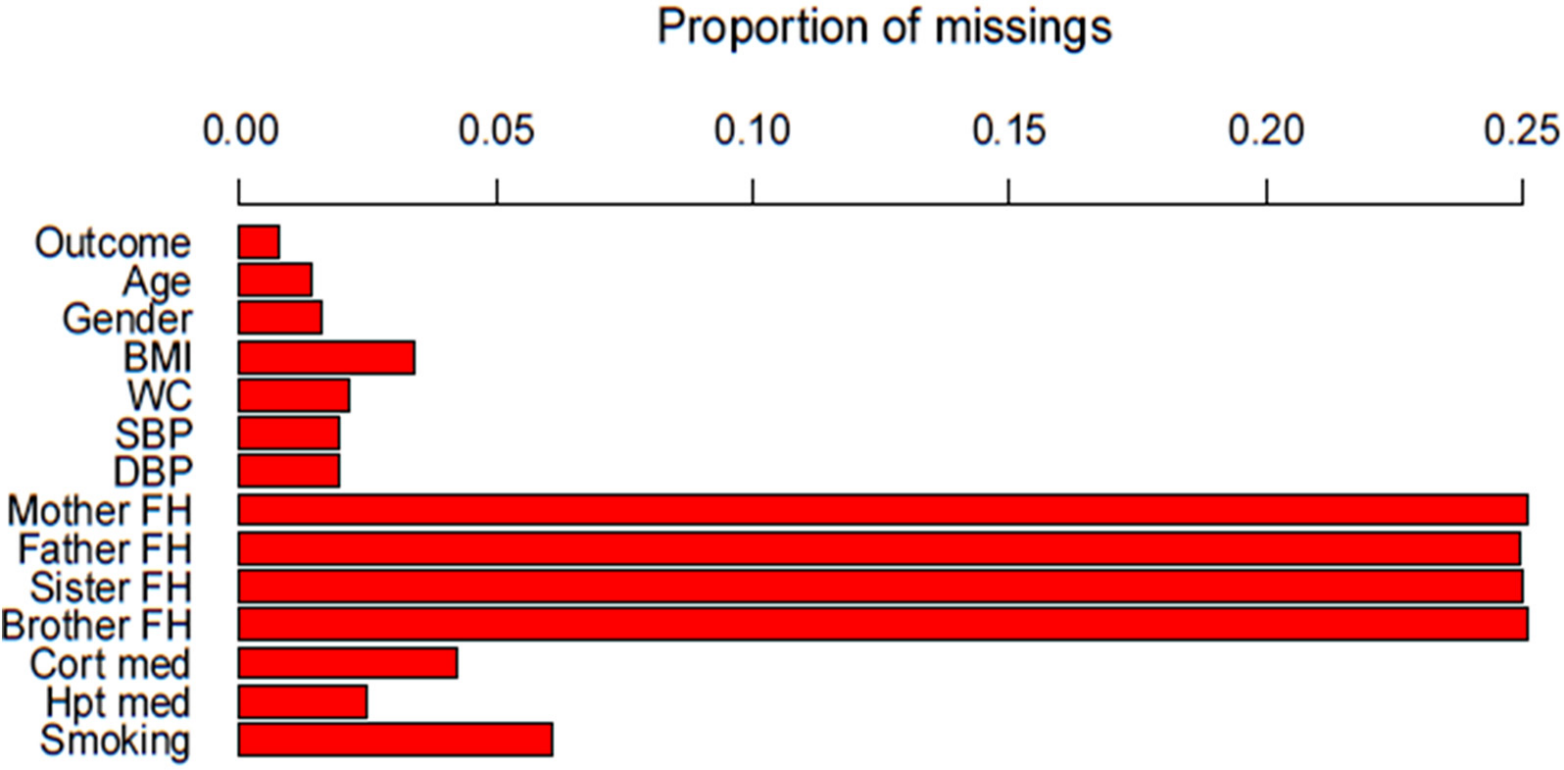
Histogram showing the proportion of missing for each variable. *BMI, Body Mass Index; WC, Waist Circumference; SBP, Systolic Blood Pressure; DBP, Diastolic Blood Pressure; FH, Family History; Cort, Corticosteroids; med, medication; Hpt, Hypertensive.

**Fig 2:**
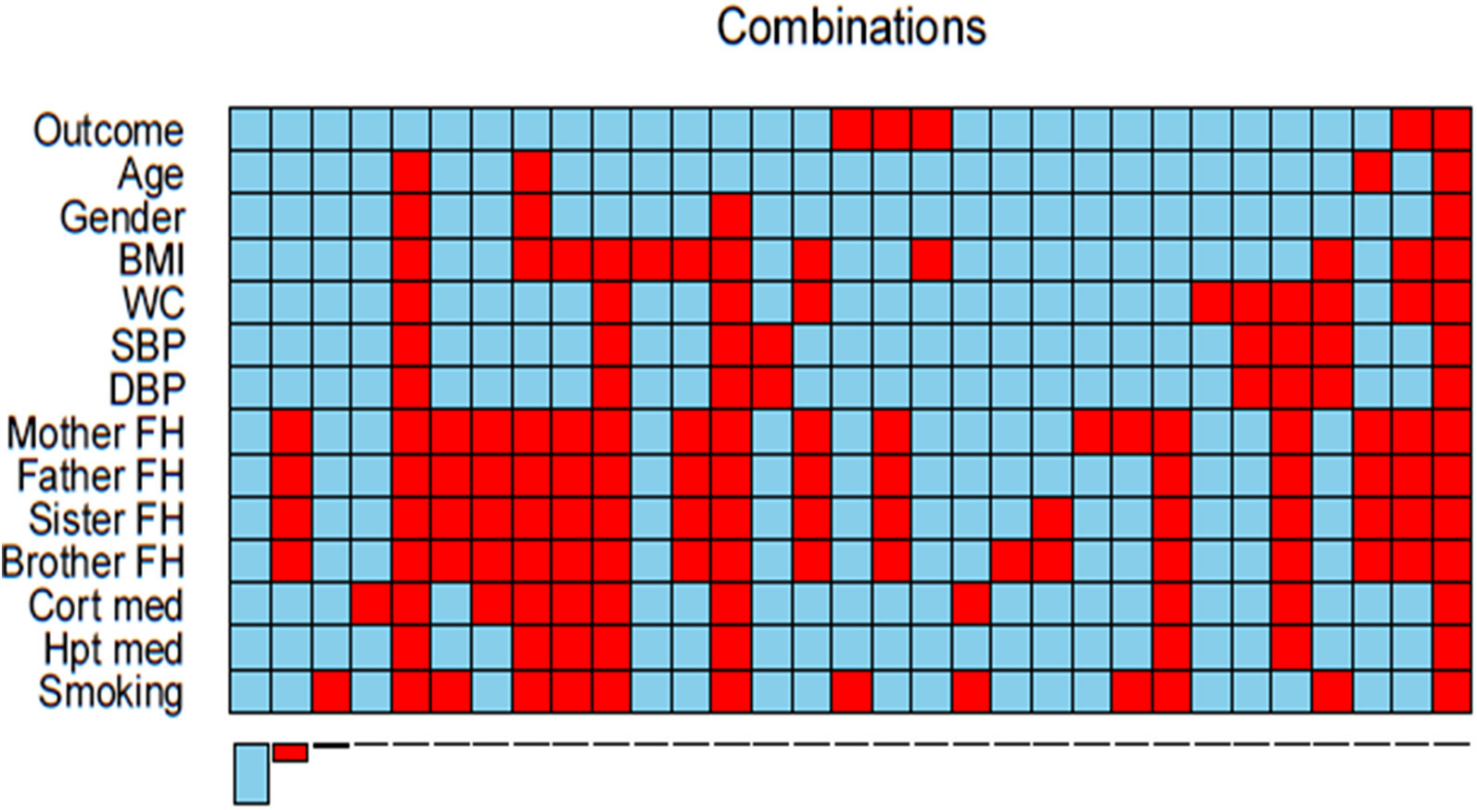
Aggregation plot showing all combinations of missing (red) and non-missing (blue) values in the variables, from the highest to lowest frequency. *BMI, Body Mass Index; WC, Waist Circumference; SBP, Systolic Blood Pressure; DBP, Diastolic Blood Pressure; FH, Family History; Cort, Corticosteroids; med, medication; Hpt, Hypertensive.

The study sample consisted of 1256 individuals, of whom 173 were excluded due to previously diagnosed diabetes. Of the final 1083 individuals, 329 (30.4%) had missing data. Table 2 summarises the number of missing values for each variable included in the 5 selected risk prediction models. Additionally, Figs 1 and 2 show the proportion and combinations of missing data respectively. Family history was the variable with the most missing data [mother (25.1%, father (24.9%), sister (25.0%), and brother (25.1%)]. The rest of the variables had a missing proportion of less than 5%, except smoking status (6.1%).

**Table 2:** Missingness analysis.

Variable	%
Outcome (prevalent diabetes)	0.7
Age	1.4
Gender	1.6
Body mass index	3.4
Waist circumference	2.1
Systolic blood pressure	1.9
Diastolic blood pressure	1.9
Mother family history	25.1
Father family history	24.9
Sister family history	25.0
Brother family history	25.1
Corticosteroid use	4.3
Hypertensive drugs	2.5
Smoking status	6.1

### Imputation

Table 3 shows the variable characteristics of the original database compared to the five methods of dealing with missing data. Pairwise deletion resulted in a significantly reduced sample size (754) while conditional mean imputation resulted in a varied length of each variables as only missing values with complete cases for every other variables were imputed. Simple imputation, stochastic regression imputation and multiple imputation all imputed all missing data allowing for a full database of 1083 individuals.

**Table 3:** Characteristics comparison of participants for the original database and five imputation methods.

	Imputation methods					
	Original	Pairwise deletion (754)	Simple (1083)	Conditional (Varied)	Stochastic (1083)	Multiple (1083)
Prevalent undiagnosed diabetes (Yes/No)	162/913	132/622	162/921	162/916	163/920	162/921
Age (years)	51.9 (15.0)	52.5 (14.6)	51.9 (14.9)	51.9 (15.0)	51.8 (15.0)	51.8 (15.1)
Body mass index (kg/m2)	29.7 (7.2)	29.6 (7.1)	29.7 (7.0)	29.7 (7.1)	29.8 (7.2)	29.8 (7.2)
Gender (Male/Female)	249/810	160/594	251/832	251/826	254/829	257/826
Systolic blood pressure (mmHg)	124.3 (20.2)	122.0 (18.7)	124.3 (20.0)	124.3 (20.2)	124.3 (20.2)	124.4 (20.4)
Diastolic blood pressure (mmHg)	76.0 (12.9)	74.7 (12.0)	76.0 (12.7)	76.0 (12.8)	76.0 (12.9)	76.1 (14.1)
Waist circumference (cm)	95.8 (15.5)	95.9 (14.9)	95.8 (15.3)	95.8 (15.4)	95.8 (15.5)	95.7 (16.9)
Hypertensive medication (Yes/No)	374/682	262/492	374/709	383/688	387/696	382/701
Using corticosteroids (Yes/No)	12/1025	5/749	12/1071	12/1050	12/1071	13/1070
Mother having diabetes (Yes/No)	124/687	114/640	124/959	124/880	182/901	165/198
Father having diabetes (Yes/No)	61/752	60/694	61/1022	61/944	73/1010	78/1005
Sister having diabetes (Yes/No)	103/709	98/656	103/980	107/897	143/940	128/955
Brother having diabetes (Yes/No)	67/744	64/690	67/1016	67/936	79/1004	87/996
Smoking status (Current/Past/No)	433/105/479	327/89/338	433/105/545	437/105/496	456/114/513	458/113/512

Imputation of the outcome, undiagnosed diabetes, was highest in stochastic regression imputation (163 individuals). Pairwise deletion saw a higher mean age (52.5 years) and lower systolic and diastolic blood pressure (122.0 mmHg and 74.7 mmHg respectively) when compared to the other imputation methods. There was no substantial difference in body mass index and waist circumference between the methods. Stochastic regression imputed higher prevalence of individuals on hypertensive medication (387 individuals), mother having diabetes (182 individuals), and sister having diabetes (143 individuals). Multiple imputation reported the highest prevalence of father (78 individuals) and brother (87 individuals) having diabetes. Variable characteristics across the five imputation datasets is shown in Table 4. Mother, father and sister family history, as well as smoking status had the most variation between the five multiple imputation datasets.

**Table 4:** Characteristics comparison of participants for five multiple imputation datasets.

		Multiple imputation datasets			
	1	2	3	4	5
Prevalent undiagnosed diabetes (Yes/No)	162/921	163/920	162/921	162/921	163/920
Age (years)	51.9 (15.1)	51.9 (15.0)	51.8 (15.0)	51.9 (15.1)	51.8 (15.0)
Body mass index (kg/m2)	29.8 (7.2)	29.8 (7.2)	29.8 (7.2)	29.8 (7.2)	29.7 (7.2)
Gender (Male/Female)	258/825	257/826	257/826	256/827	258/825
Systolic blood pressure (mmHg)	124.5 (20.4)	124.5 (20.3)	124.4 (20.3)	124.5 (20.4)	124.3 (20.3)
Diastolic blood pressure (mmHg)	76.1 (12.8)	76.1 (12.8)	76.2 (13.3)	76.1 (12.9)	76.1 (12.9)
Waist circumference (cm)	95.8 (15.9)	95.7 (15.5)	95.8 (15.5)	95.8 (15.4)	95.7 (15.5)
Hypertensive medication (Yes/No)	383/700	378/705	381/702	382/701	384/699
Using corticosteroids (Yes/No)	13/1070	12/1071	12/1071	13/1070	13/1070
Mother having diabetes (Yes/No)	157/926	168/915	155/928	179/904	164/919
Father having diabetes (Yes/No)	71/1012	72/1011	83/1000	78/1005	84/999
Sister having diabetes (Yes/No)	132/951	130/953	121/962	125/958	134/949
Brother having diabetes (Yes/No)	87/996	88/995	83/1000	88/997	88/995
Smoking status (Current/Ex/No)	464/110/509	455/118/510	459/115/509	452/113/518	460/111/512

### Model performance

Most notably, model performance following pairwise deletion deviated from the model performance from other imputation methods. The discrimination was lower in all five models, however calibration was better in the Cambridge Diabetes Risk model [1.81 (1.09–2.52)]. Overall, although not large differences, simple imputation yielded the highest C-statistic for four of the five models; the Cambridge Diabetes Risk model [0.69 (0.65–0.73), vs. 0.67 (0.62–0.72)], Kuwaiti Risk model [0.70 (0.66–0.74), vs. 0.68 (0.63–0.73)], Omani Diabetes Risk model [0.67 (0.63–0.71) vs. 0.65 (0.61–0.69)] and Rotterdam Predictive model [0.65 (0.61–0.70) vs. 0.64 (0.59–0.69)]. Multiple imputation only yielded the highest C-statistic for the Rotterdam Predictive model [0.65 (0.61–0.70), which were matched by simpler imputation methods. Table 5 details the indifference in model performance across the five datasets produced through multiple imputation.

**Table 5:** Overview of the performance of the undiagnosed diabetes risk prediction models across the five multiple imputation datasets.

Multiple imputation datasets		1	2	3	4	5
Cambridge	E/O (95% CI)	2.17 (1.35–2.99)	2.13 (1.40–2.87)	2.15 (1.49–2.81)	2.18 (1.34–3.01)	2.16 (1.46–3.86)
Diabetes Risk model	Brier score	0.190	0.188	0.186	0.190	0.190
	Yates slope	-1.451	-1.435	-1.433	-1.454	-1.434
	C-statistic (95% CI)	0.68 (0.64–0.72)	0.68 (0.64–0.72)	0.69 (0.65–0.73)	0.68 (0.64–0.73)	0.69 (0.64–0.73)
Kuwaiti Risk model	E/O (95% CI)	0.83 (0.42–1.24)	0.82 (0.40–1.23)	0.82 (0.45–1.19)	0.83 (0.41–1.24)	0.82 (0.44–1.19)
	Brier score	0.124	0.124	0.122	0.123	0.123
	Yates slope	-0.563	-0.558	-0.496	-0.542	-0.509
	C-statistic (95% CI)	0.69 (0.65–0.73)	0.69 (0.64–0.73)	0.70 (0.66–0.74)	0.69 (0.65–0.73)	0.69 (0.65–0.74)
Omani Diabetes	E/O (95% CI)	1.55 (0.76–2.33)	1.54 (0.72–2.37)	1.52 (0.87–2.17)	1.57 (0.78–2.37)	1.54 (0.80–2.29)
Risk model	Brier score	0.154	0.156	0.149	0.155	0.153
	Yates slope	-1.211	-1.232	-1.151	-1.214	-1.174
	C-statistic (95% CI)	0.65 (0.61–0.69)	0.64 (0.60–0.68)	0.66 (0.62–0.70)	0.65 (0.61–0.70)	0.66 (0.61–0.70)
Rotterdam	E/O (95% CI)	0.66 (0.57–0.75)	0.65 (0.58–0.72)	0.65 (0.57–0.74)	0.66 (0.57–0.75)	0.65 (0.57–0.74)
Predictive model	Brier score	0.126	0.127	0.126	0.127	0.127
	Yates slope	0.486	0.539	0.526	0.479	0.461
	C-statistic (95% CI)	0.65 (0.60–0.69)	0.65 (0.61–0.70)	0.65 (0.60–0.70)	0.65 (0.60–0.69)	0.65 (0.60–0.69)
Simplified Finnish	E/O (95% CI)	0.35 (0.17–0.52)	0.34 (0.16–0.52)	0.35 (0.17–0.53)	0.35 (0.17–0.52)	0.34 (0.16–0.52)
Diabetes Risk model	Brier score	0.133	0.134	0.133	0.133	0.134
	Yates slope	-0.032	-0.068	-0.048	-0.026	-0.050
	C-statistic (95% CI)	0.66 (0.62–0.71)	0.66 (0.62–0.70)	0.66 (0.62–0.70)	0.66 (0.62–0.71)	0.66 (0.62–0.70)

The pattern of the overall calibration (E/O) did not vary substantially across imputation methods. Uniformly, all imputation methods resulted in the Cambridge and Omani risk models overestimating diabetes risk, while the others showed underestimation. Other performance measures across subgroups, shown in Table 1, did also not show significant differences between imputation methods. When recalibration was performed, all models across all imputation techniques had an improved agreement between predicted and observed rates (Table 1).

## Discussion

The suggested imputation method for the handling of missing data is a hot topic, with strong advocators for multiple imputation and those who propose that simple techniques can be just as effective. Several studies have been done to determine the effect of several imputation methods on the predictive performance of risk models, however these have been largely contradicting. Donders *et al* [1] performed a simulation study in an attempt to illustrate that single imputation yields unbiased estimates with too narrow confidence intervals and multiple imputation indeed yields unbiased estimates with correct standard errors. Both single and multiple imputation produced unbiased estimates of association, and the conclusion was that despite single imputation appearing more precise, multiple imputation produces less bias and more precise results. Alternately, a study by van der Heijden *et al* [15] concluded that the models fitted using the indicator method, a simple method of dealing with missing data, showed higher regression coefficients and predictive accuracy when compared to the models derived from the imputation methods. As confirmed in this study, we did not observe large differences between the models obtained after single unconditional, single conditional and multiple imputation of the missing data. Deletion of individuals with missing data resulting in an expected reduced discriminatory ability of the models. Model calibration was improved across all areas when recalibration was performed. This however, has no influence on the imputation techniques or the discriminatory ability of the models.

What should be noted is that most studies comparing imputation techniques start with a complete data set and introduce missing data to set variables. Our study made use of an existing database which already included missing data on a number of variables. This results in the true underlying value of the missing data being unknown, as well as the true regression coefficients and predictive accuracy of each variable. This lack of reference criterion can be limitation in a study. However, the use of existing databases should be encouraged as this is more translatable to medical research outside of a controlled setting.

Despite recent advances in understanding missing data and imputation methods, most researchers still report deletion, perhaps because of a lack of adequate guidelines for handling missing data. What should be encouraged is the use of more than one method, the results compared and a preferred approach chosen and defended. When data are missing on several variables it is important to use some procedure that imputes them all together, rather than one variable at a time. This ensures that the imputed data are related to each other in the same way as those data that are observed.

## Conclusion

This study aimed to compare the performance results of undiagnosed diabetes risk prediction models across multiple imputation techniques. The results showed a lower model performance when deletion is used to deal with missing data and little difference between simple and more complex methods on the effect of risk prediction model performance. Missing data is an important aspect of predictive research and needs to be handled correctly. Imputation, specifically more complex and time-intensive imputation, can often be avoided by researchers due to preconceived complexity. Simpler imputation methods that allow for similar or better predictive performance are easy to undertake and should encourage researchers of all levels to limit the use of deletion of individuals with missing data. The negligible difference in model performance between simple and multiple imputation allows for the recommendation of single imputation for handling missing data in undiagnosed diabetes predicative research.
